# Influencing factors and mechanisms of action on the participation intentions of cryptocurrency investment fraud victims—A quantitative examination from the perspective of the theory of planned behavior

**DOI:** 10.1371/journal.pone.0339989

**Published:** 2026-02-25

**Authors:** Jianbo Wang, Liang Deng

**Affiliations:** School of Law, Capital University of Economics and Business, Beijing, China; Indian Institute of Management Sambalpur, INDIA

## Abstract

With the rise of cryptocurrency, using cryptocurrency investments to conduct fraud has become a common criminal tactic. Drawing on survey data from 287 victims in China, this study explored the determinants and mechanisms of investment intention among victims of cryptocurrency investment scams. Based on the TPB and using SEM, we identified three main findings: (1) Investment attitude and perceived behavioral control have a significant positive impact on victims’ intention to invest. (2) Risk-seeking personality traits, laws and regulations, investment education, and fraud cases exposure not only directly affect this intention but also influence it indirectly through investment attitude and perceived behavioral control. (3) Subjective norms have a limited impact on investment intention. These conclusions suggest a challenge to the traditional TPB. The decentralized nature of cryptocurrency may make victims rely more on personal judgment than social influence. This finding expands the applicability of the TPB. It also provides a basis for developing targeted fraud prevention systems.

## 1. Introduction

Cryptocurrency generally refers to blockchain-based digital currencies maintained on decentralized networks [1], such as Bitcoin, Ethereum, etc. They are characterized by decentralization, globalization, and strong concealment. Cryptocurrency investment is a new type of investment method that conducts transactions through internet platforms, using cryptocurrency as the medium of exchange. Its high-risk and high-reward nature has attracted the attention of many investors. However, due to large market volatility and lack of regulation, the cryptocurrency market has become a hotbed for criminal activities [2]. In recent years, cryptocurrency investment fraud has become a common criminal behavior. Criminals often use cryptocurrencies or air coins as investment objects, attracting investors with the promise of fictitious high returns. Then defraud them of their funds through various means. These fraudulent behaviors are highly deceptive and confusing, making it difficult for investors to identify and prevent them.

The Federal Bureau of Investigation (FBI) disclosed that the losses from cryptocurrency fraud schemes soared to $3.96 billion in 2023, an increase of 53% from the previous year. Investment fraud has become the main form of cryptocurrency-related crimes, accounting for nearly 71% of all cryptocurrency losses. In August 2024, the Australian Securities and Investments Commission (ASIC) announced that it had dismantled more than 600 cryptocurrency scams in the past 12 months, and more than 7,300 phishing and fraudulent websites had been closed. In November 2024, Korean police arrested 215 people suspected of involvement in a cryptocurrency investment scam that defrauded 15,000 people of 1.65 billion yuan, claiming it was the largest cryptocurrency investment scam in South Korea’s history.

In the past period, cryptocurrencies have been subjected to stricter scrutiny and regulation worldwide. The UK Financial Conduct Authority (FCA) approved only 4 out of 35 cryptocurrency company registration applications received during the 2023−24 fiscal year, with over 87% of applications rejected for not meeting approval standards. In October 2022, the European Parliament’s Committee passed the “Markets in Crypto-Assets Regulation” (MiCA), which has clear and strict regulatory systems for crypto exchanges and other intermediary service providers, as well as stablecoin issuers and other crypto projects, forming a unified crypto regulatory system across the EU countries with fragmented regulatory policies. The Korean Financial Supervisory Service will inspect the first batch of virtual asset companies after the implementation of the “Virtual Asset Protection Act” since July. The Qatar Financial Centre (QFC) has released a comprehensive digital asset regulatory framework, including the tokenization process, legal recognition of property rights for tokens and their underlying assets, custody arrangements, transfer and exchange, and regulations on the legal recognition of smart contracts. Nigeria has issued stricter new crypto regulations to combat digital currency exchanges that do not comply with its rules. China is one of the earliest countries in the world to regulate cryptocurrencies, with strict regulatory measures in place. Since 2013, it has continuously issued a series of laws and regulations, denying the legality of cryptocurrencies as currency or securities for trading and free exchange within its borders from both a systemic and enforcement level.

Even under strict regulation, cryptocurrency investment fraud remains rampant. The reasons why victims fall for these scams are worth exploring. This paper addresses two key questions: (1) What factors influence victimization? (2) How do these factors affect victims?

Our study differs from prior literature in three ways: First, we applied the TPB to victim analysis. This theory helped analyze internal and external factors. A victim’s participation in cryptocurrency investment fraud is a conscious, planned behavior, and participation intention plays a significant role in the process of behavior implementation. Second, this paper explored the dynamic process of victim decision-making in the context of cryptocurrency fraud. By analyzing personality traits and external factors, it elucidated the chain of behaviors leading to victim participation. This perspective shifts the research focus from “passive victimization” to “active decision-making”, thereby uncovering the “intention-driven” mechanism behind victimization. The framework emphasizes the behavioral rationality of victims-even if it is limited rationality-within an environment of information asymmetry. In doing so, it challenges the stereotype of the “irrational victim”. Third, we used quantitative research methods. We employed SEM to test our hypotheses. This model analyzed the factors influencing Chinese victims. It also revealed their underlying mechanisms.

Our study has practical implications: (a) For victims, our results clarify how to reduce scam risk at an individual level. (b) For policymakers, we highlight the need to strengthen efforts. This includes improving regulatory communication and deepening investment education.

## 2. Literature review

### 2.1. Research on issues related to cryptocurrency fraud crimes

Research on cryptocurrency crimes involves related legal norms, types of crimes, nature of crimes, causes of crimes, and risk prevention. Specifically, research on cryptocurrency fraud crimes focuses on the following aspects:

1. Types of cryptocurrency fraud crimes: After analyzing a large number of cryptocurrency fraud cases, Baum identified four main types of cryptocurrency fraud: Ponzi schemes, fake ICOs (Initial Coin Offerings), pump-and-dump schemes, and trading service fraud [3]. Trozze et al. systematical study found that Ponzi schemes and (synonymous) high yield investment programmes were most discussed across all literature, and provided a comprehensive definition of this fraudulent activities to promote the development of cryptocurrency industry standards and help prevent cryptocurrency fraud [4].
2. Risks and regulatory countermeasures caused by cryptocurrency fraud: Marijan et al. analyzed that bitcoin and other cryptocurrencies are still suitable for fraudulent activities in financial markets and emphasize the importance of institutions in reducing potential risks. [5]. Tiwari et al. examined the risks associated with fraud using initial token offerings and revealed key lessons for investors in terms of proactive steps that can be taken to protect themselves from being victims, for issuers to ensure awareness and take steps to secure investors’ trust, and for regulators to promote a safe environment. [6]. Kerr et al. described the risks of cryptocurrency fraud and analyzed the financial performance of cryptocurrencies, noting that the risks of investing in cryptocurrencies are much higher than traditional stock market investments [7].
3. Technical detection methods proposed based on the principles of cryptocurrency fraud: Nolasco and Vaughn studied cryptocurrency fraud using the convenience theory, identifying favorable conditions that can be exploited by criminals in cryptocurrency crimes [8]. Sureshbhai et al. proposed a blockchain-based SA framework for detecting cryptocurrency fraud from a deep learning perspective, providing technical early warning for cryptocurrency fraud [9]. Ravindranath et al. used oversampling techniques for Ethereum fraud detection through data cleaning, correlation analysis, data splitting, and exploratory data analysis, integrating models [10]. Abdulkadhim et al. used recurrent neural networks for cryptocurrency-related fraud detection and provided a taxonomy of crypto-forensics and forensic blockchain [11]. Agarwal et al. also introduced an architecture integrating Artificial Intelligence (AI) and blockchain technology to investigate and prevent instances of cryptocurrency fraud [12].

Currently, there are more articles that provide an overall study of cryptocurrency crimes, but fewer that specifically analyze cryptocurrency fraud crimes. The existing research mainly focuses on the patterns of cryptocurrency fraud, risk regulation, and detection warnings, with less research on how to prevent cryptocurrency fraud from the victim’s perspective. The traditional research on fraud victims mainly focuses on the following aspects: first, the analysis of victim characteristics, including demographic characteristics (such as age, gender, education level), psychological traits (such as trust tendency, risk perception, cognitive bias), and socio-economic background; second, the study of fraud methods, analyzing the technical means of fraudsters (such as fake platforms, false promises) and social engineering strategies (such as emotional manipulation, authority camouflage); third, the impact on victims, discussing the economic losses, psychological trauma, and social stigmatization of victims.

### 2.2. Application of the theory of planned behavior in the field of crime research

Ajzeni proposed the Theory of Planned Behavior (TPB) based on the Theory of Rational Action [13]. TPB suggests that people engage in behaviors through deliberate planning, and behavioral intention is a crucial motivational factor influencing an individual’s performance of a certain behavior. Behavioral intention is influenced by personal attitudes, external subjective norms, and perceived behavioral control, which are the direct causes driving behavior and key indicators for predicting the strength of behavior occurrence. Therefore, it is necessary to explore individual participation in behavior from the perspectives of internal mechanisms of individual behavior and social structures. Current research shows that scholars and practitioners have conducted some beneficial discussions on the application of TPB in crime research. The application of TPB in the field of crime can be divided into two categories:

1. The direct impact of the three variables in TPB on illegal and criminal behavior. María del Mar et al. constructed an action system model, which distinguishes crimes based on behavioral indicators and establishes a relationship between the actions of the offender at the crime scene and their characteristics [14]. Curtis et al. found that both self-control and perceived behavioral control have an additive effect on predicting plagiarism [15]. Skrzypiec, through empirical testing, found that attitudes, subjective norms, perceived control, and negative effects are important factors related to adolescent criminal intentions, but moral norms and reputation enhancement play a more distant role [16].
2. Analyzing how other factors influence illegal and criminal intentions through the three variables in TPB. Sari and Arslantas concluded by data analysis that males, oppression, indifferent family attitudes, frequent family debates, exposure to violent content, father’s criminal conviction, low parental education level, and adolescents with delinquent behavior have a higher risk of criminal behavior [17]. Bin et al. analyzed the impact of various transportation policies on female behavior, with CCTV, accessible alarms, and more police presence appearing to be more effective in reducing the number of sexual crimes [18]. Willison and Warkentin suggests that when investigating violations of computer security regulations, it is important to consider the thought processes of potential offenders and how these processes are influenced by the organizational environment prior to deterrence [19].

Traditionally, when applying the Theory of Planned Behavior (TPB) to criminal behavior, research has focused more on direct paths to participation. It has also rarely involved subjects with strong technical and decentralized characteristics. This paper applies the TPB to the cryptocurrency investment field, expanding its applicability. Furthermore, it investigates the indirect paths among influencing factors, enriching the discussion of the TPB’ s underlying mechanisms.

## 3. Research design

### 3.1. Analytical framework

**Fig 1:**
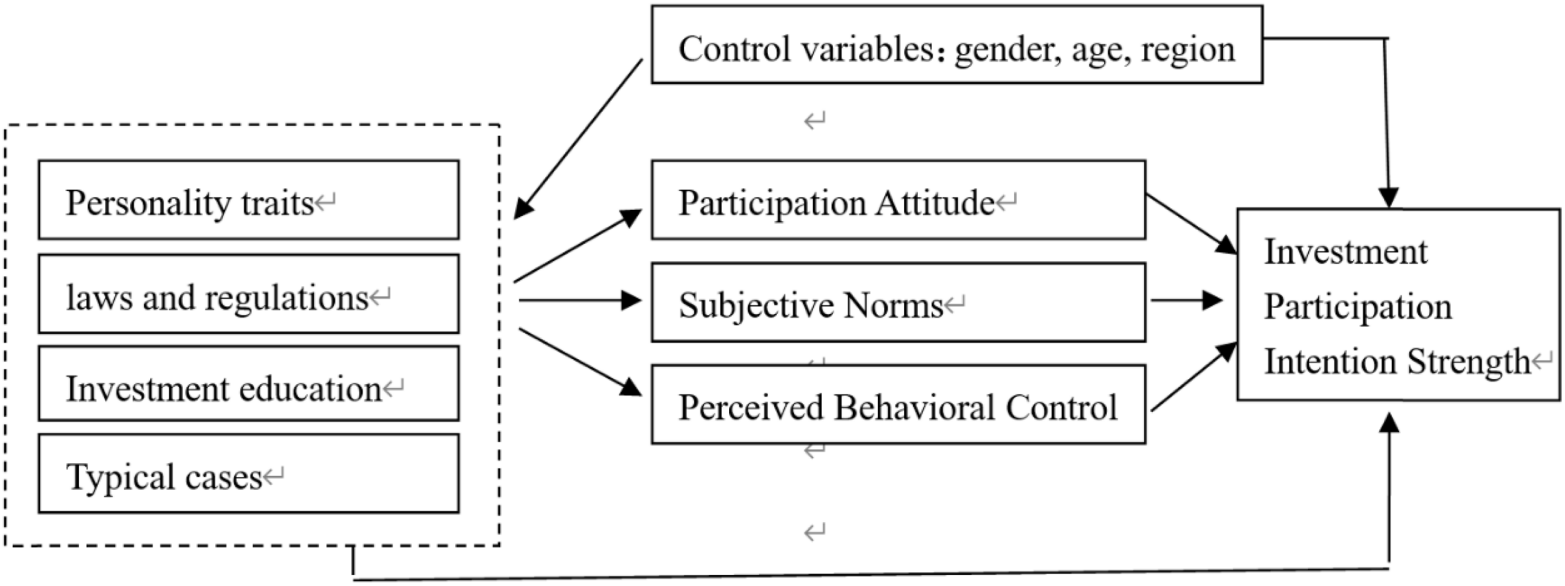
Analytical framework for factors affecting the strength of victims’ participation intention in cryptocurrency investment fraud from the perspective of the theory of planned behavior.

To investigate the factors and mechanisms influencing the intensity of investment intention among victims of cryptocurrency investment scams, this paper proposed several hypotheses based on prior research and the fundamental principles of the TPB. SEM was then employed for statistical analysis. This paper argues that after controlling for factors such as gender, age, and region, the strength of victims’ participation intention in cryptocurrency investment fraud is not only directly influenced by personality traits and external factors (investment education, laws and regulations, typical cases), but also indirectly affected by these factors through mediating variables such as investment attitude, subjective norms, and perceived behavioral control (In Fig 1). Taking personality traits as an example, individuals with traits such as confidence and risk preference often have a stronger inclination to participate in investments [20]. Therefore, personality traits will directly affect individual participation behavior. At the same time, individuals with these traits may have a stronger investment participation attitude and, to some extent, change their cognition and self-evaluation of their ability to participate in investments. That is, personality traits also affect the strength of victims’ participation intention in cryptocurrency investment fraud through investment attitude, subjective norms, and perceived behavioral control.

The study makes the following hypotheses:

**H1a.** Personal traits have a direct impact on participation intention.
**H1b.** Personal traits indirectly affect participation intention by participation attitude, subjective norms, and perceived behavioral control.
**H2a.** Regulatory policies have a direct impact on participation intention.
**H2b.** Regulatory policies indirectly affect participation intention by participation attitude, subjective norms, and perceived behavioral control.
**H3a.** Investment education has a direct impact on participation intention.
**H3b.** Investment education indirectly affects participation intention by participation attitude, subjective norms, and perceived behavioral control.
**H4a.** Typical cases have a direct impact on participation intention.
**H4b.** Typical cases indirectly affect participation intention by participation attitude, subjective norms, and perceived behavioral control.
**H5.** Participation attitudes have a direct impact on participation intention.
**H6.** Subjective norms have a direct impact on participation intention.
**H7.** Perceived behavioral control has a direct impact on participation intention.

### 3.2. Measurement model

In this study, the dependent variable “victims’ participation intention in cryptocurrency investment fraud” is a continuous variable, while the independent variables are mostly discrete. Therefore, this paper employs a multiple linear regression model for the analysis. The regression model is expressed as: Y = α_0_ + α_1_X_1_ + α_2_X_2_ + … + α_n_X_n_ + ε, where Y represents the dependent variable, X_1_, X_2_ … X_n_ represent the independent variables, α_0_ is the constant term, α_1_, α_2_ … α_n_ are the regression coefficients for each independent variable, which indicate the dynamic change in the dependent variable for each unit change in the independent variable, assuming other independent variables remain constant, and ε represents the random error.

To further explore the interaction mechanism of factors affecting the strength of victims’ participation intention in cryptocurrency investment fraud, this paper uses a Structural Equation Model (SEM) for analysis. SEM integrates factor analysis and path analysis, simultaneously examining the relationships between observed variables, latent variables, and residual terms in the model. It allows for the determination of the direct, indirect, and total effects of independent variables on the dependent variable. The SEM consists of a measurement model and a structural model, with the specific model structure as follows:


X=ΛXξ+δ (1)

Y=ΛYη+ε (2)

η=αη+Γξ+ζ (3)

Where, equations (1) and (2) are the measurement models, X is the exogenous variable, Y is the endogenous variable, ΛX and ΛY are the factor loading matrices, δ and ε are error terms, equation (3) is the structural model, α is the path coefficient indicating the relationship between endogenous latent variables, η is a latent variable corresponding to the endogenous variable, Γ is the path coefficient indicating the influence of exogenous latent variables on endogenous latent variables, ξ is a latent variable corresponding to the exogenous variable, and ζ is the residual term of the structural equation, reflecting the unexplained part of the equation. The measurement model describes the relationship between latent variables and their indicators, while the structural model describes the relationships between the latent variables.

### 3.3. Participants

The study adopted an anonymous sampling method for victims all from China. With the assistance of police officers handling cryptocurrency investment fraud cases, the author distributed a questionnaire link to the victims. Using this method, a total of 322 questionnaires were collected, of which 287 were valid, resulting in an effective response rate of 89%. In path analysis and structural equation modeling studies, it is recommended that the sample size for each variable range from 10 to 20 individuals [21]. Considering the eight variables in this study, the sample size of 287 individuals calculated using the Cochran formula is considered sufficient (see Appendix 1 in S1 File).

### 3.4. Variable design

The connotations and measurement methods of the dependent variable, independent variables, and control variables are detailed in Table 1. The victims used a five-point Likert scale ranging from 1 (strongly disagree) to 5 (strongly agree), to answer eight items related. A total of 35 dimensions were set up. In addition to the three components of the TPB—investment attitude, subjective norms, and perceived behavioral control—this study also considers the following four aspects based on previous research findings that influence the intention of victims to participate in cryptocurrency investment scams: First, the impact of personality traits on the investment intention of victims. Scholars such as Mc Inish have early explored the relationship between personality traits like extroversion, agreeableness, and even humility, anxiety, with investment behavior [22]. Mayfield et al. analyzed investors’ personality traits based on the Five-Factor Model (OCEAN) and found that investors’ neuroticism and extroversion were correlated with their short-term investment intention, while agreeableness did not have a significant impact on investment intention [23]. Secondly, the influence of investment education level on the investment decisions of victims. Hastings et al. emphasized that targeted and timely financial education is more effective in intervening in investment decisions [24]. Lusardi and Mitchell also found that the timeliness, depth, and intensity of financial education are key to the success of financial education interventions [25]. Hira and Cäzilia found that financial education in the workplace can enhance employees’ financial literacy and improve the rationality of their investment decisions [26]. Thirdly, the relationship between legal knowledge and the victimization of fraud. Wu et al. proposed that strengthening the legal knowledge learning and legal concept cultivation for retired elderly people is important to improve their anti-fraud awareness [27]. Lu pointed out that enhancing legal education and anti-fraud awareness is crucial for the prevention of telecommunication fraud crimes from the source [28]. Fourth, the impact of victims’ cognition and assessment of cryptocurrency investment fraud cases on their intention to participate. Read and Loewenstein found in their earlier research that when investors experience an investment impulse, they automatically trigger certain psychological responses to assess whether there are limiting factors, thus determining whether to engage in the investment behavior [29]. Mehrabian and Russell added the concept of “organism” to the stimulus-response theory, forming the stimulus-organism-response model, where “organism” represents a person’s internal cognitive and emotional processes [30].

**Table 1:** Measurement of Various Variables.

Variable Type	Variable Name	Connotation	Questionnaire Abbreviations
Dependent Variable	Investment Participation Intention Strength	The strength of the victim’s intention to participate in investments	PI1 PI2 PI3
Independent Variable	Personality Traits	Psychological characteristics of victim	PT1 PT2 PT3 PT4 PT5
	Laws and Regulations	The extent of the victim’s understanding of the regulatory framework for cryptocurrencies and the legal policies related to the involved crimes.	RP1 RP2 RP3 RP4 RP5
	Investment Education	The situation of the victim receiving education on cryptocurrency investment.	IE1 IE2 IE3 IE4
	Typical Cases	The cognitive impact of experiencing or witnessing investment fraud cases on the victim	TC1 TC2 TC3 TC4
Mediating Variable	Participation Attitude	Assessment of cryptocurrency investment on the Degree of Victim Needs Satisfaction	PA1 PA2 PA3 PA4 PA5
	Subjective Norms	The opinions and attitudes of important others around the victim towards their participation in a particular investment	SN1 SN2 SN3 SN4 SN5
	Perceived Behavioral Control	The degree to which the victim believes they have the ability to invest and control it	PBC1 PBC2 PBC3 PBC4
Control Variable	Gender	Male = 1; Female = 2	
	Age	Youth (20–40 years old) = 1; Middle-aged (40–60 years old) = 2; Elderly (above 60 years old) = 3	
	Region	Coastal = 1; Inland = 2	

## 4. Empirical results and analysis

### 4.1. Basic information on the strength of victims’ participation intention in cryptocurrency investment fraud

A summary of the survey respondents’ general demographic characteristics is shown in Table 2. The profile represents the participants’ age, gender, and region.

**Table 2:** Demographic profile.

Characteristics	Category	Number	Percent (%)
**Age**	20 to 40 years old	89	31.01
	41 to 60 years old	93	32.40
	>60 years old	105	36.59
**Gender**	Female	140	48.78
	Male	147	51.22
**Region**	Inland	155	54.01
	Coastal	132	45.99
Total		287	100

According to the definition in the “China Marine Statistical Yearbook,” China’s coastal region includes 14 provincial-level administrative units: Liaoning, Hebei, Tianjin, Shandong, Jiangsu, Shanghai, Zhejiang, Fujian, Guangdong, Guangxi, Hainan, and so on.

Based on the survey data, we have plotted the average strength of victims’ participation intention in cryptocurrency investment fraud across different genders, age groups, and regions.

**Fig 2:**
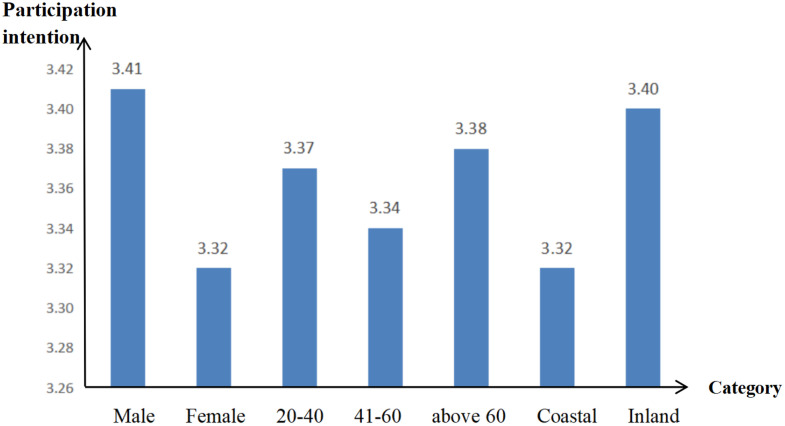
Bar chart of the strength of victims’ participation intention in cryptocurrency investment fraud.

From Fig 2, it can be observed that the overall level of victims’ participation intention in China is relatively high. Specifically: There is a significant difference in participation intention between men and women, which is related to the substantial differences in personality traits between the two genders [31]. The investment intention in inland is much higher than in coastal, this is likely because coastal economies are more developed, leading to stronger learning capabilities, better information access, and consequently, a stronger awareness of fraud prevention. In terms of age, victims aged 20–40 and over 60 show slightly higher intention than those aged 40–60, this may be because middle-aged people are more mature and prudent, considering problems more carefully and thoroughly, which lowers their risk of being scammed.

### 4.2. Reliability test

To test the reliability and validity, we examined factor loadings, Cronbach alphas and composite reliability. The results of the Confirmatory Factor Analysis (CFA) indicate that all latent variables demonstrate convergent validity and internal consistency. All standardized factor loading coefficients were above 0.73, with most exceeding 0.8 and some even reaching over 0.9, reflecting a strong association between the latent variables and their measurement indicators. The Average Variance Extracted (AVE) values for each construct ranged from 0.609 to 0.760, all surpassing the threshold of 0.5. The Composite Reliability (CR) values fell within the interval of 0.884 to 0.941, with the “laws and regulations” construct demonstrating the highest internal consistency (CR = 0.941). The Cronbach’ s alpha coefficients for all constructs were highly consistent with their CR values and all reached an excellent level. Collectively, these indicators confirm that the convergent validity and reliability of the measurement instrument meet the rigorous standards required for the research, thereby validating the stability and reliability of the measurement model (see Appendix 2 in S1 File).

Meanwhile, the factor analysis results (Appendix 3 in S1 File) revealed a clear seven-factor structure. The loading coefficients of all measurement items on their corresponding factors exceeded 0.778, demonstrating excellent convergent validity. The items were independent of each other, with no significant cross-loadings observed. The communalities ranged from 0.651 to 0.873, indicating that the seven extracted factors sufficiently explained the variance in each observed variable. The rotated factor structure was more balanced, with the variance explained by each factor distributed between 9.456% and 12.851%. The cumulative variance explained reached 78.169%, far exceeding the commonly accepted 60% threshold in social science research. The two constructs of “Laws and regulations” and “Personality traits” accounted for the largest proportion of variance. This outcome is highly consistent with theoretical expectations, providing strong confirmation of the measurement tool’s construct validity and the discriminant validity of its constructs. The scale’s structure is robust, and the boundaries between constructs are clear, laying a solid foundation for the subsequent empirical analysis.

In Appendix 4 in S1 File, the high KMO value of 0.920 indicates strong partial correlations among the variables. Simultaneously, the results of Bartlett’s Test of Sphericity show an approximate chi-square value of 7310.113 (df = 496, p < .001). This statistically significant result (p = .000) decisively rejects the null hypothesis that the correlation matrix is an identity matrix. This implies the presence of significant multivariate correlations among the observed variables, providing a solid data structural foundation for conducting Exploratory Factor Analysis (EFA).

In Appendix 5 in S1 File, the results of the discriminant validity test indicate that the constructs demonstrate good discriminant validity, as evidenced by the correlation matrix. The values on the diagonal of the matrix (representing the square root of the AVE for each construct) are all higher than the corresponding off-diagonal elements in their respective rows and columns, confirming the distinctiveness among the constructs. A moderate negative correlation was found between personality traits and regulatory policies (r = −0.260), while a strong positive correlation was observed between regulatory policies and investment education (r = 0.500). The strongest negative correlation was found between typical cases and investment education (r = −0.522), and the highest correlation was between attitude toward participation and perceived behavioral control (r = 0.559). Overall, most correlation coefficients among the constructs fall within a reasonable range, reflecting both theoretical associations and the uniqueness of each measurement dimension. Subjective norms showed generally low correlations with other variables, indicating its independence from the other constructs. These findings collectively confirm that the dimensions of the measurement model maintain both theoretical coherence and sufficient discriminant validity, laying a solid foundation for the structural model analysis.

### 4.3. Common method bias

In order to examine the potential common method bias, we adopted the one-factor method recommended by Slater et al. [32]. The results of seven-factor model demonstrate an excellent model fit. Its Comparative Fit Index (CFI) and Tucker-Lewis Index (TLI) values reached 0.981 and 0.979, respectively far exceeding the 0.95 threshold for a good fit. The Root Mean Square Error of Approximation (RMSEA) was 0.053, and the Standardized Root Mean Square Residual (SRMR) was only 0.029, both of which are within the ideal range. In contrast, the more parsimonious comparison models exhibited significantly poorer fit. The fit indices for the five-factor model had already markedly decreased (CFI = 0.622, TLI = 0.776, RMSEA = 0.116), and the more simplified models showed a trend of further deterioration, with the single-factor model yielding the worst fit (CFI = 0.453, TLI = 0.419). These findings robustly validate that the seven-dimensional structure of the research constructs possesses clear discriminant validity and independence. Although correlations exist among the constructs, they cannot be merged. The CFA results are highly consistent with the findings from the Exploratory Factor Analysis (EFA), which further confirms the construct validity of the measurement instrument and the soundness of the theoretical model (see Appendix 6 in S1 File).

### 4.4. Multiple linear regression analysis of factors affecting the strength of victims’ participation intention in cryptocurrency investment fraud

Using the participation intention, personality traits, laws and regulations, investment education, typical cases, participation attitude, subjective norms, and perceived behavioral control as latent variables, with gender, age, and region as control variables, a multiple linear regression analysis was conducted using SPSS 20.0 statistical software. The regression results are presented in Table 3.

**Table 3:** Multiple linear regression analysis of factors affecting the strength of victims’ participation intention in cryptocurrency investment fraud.

Variable		Non-standardized coefficients		standardized coefficients	*t*	*p*	Collinearity Diagnostics	
		*B*	S.E.	*Beta*			VIF	Tolerance
**Participation Intention**	Constant	4.481	0.234	–	19.111	0.000**	–	–
	Gender	0.053	0.065	0.033	0.805	0.421	1.043	0.959
	Age	0.025	0.039	0.025	0.639	0.523	1.009	0.991
	Region	0.017	0.065	0.010	0.257	0.797	1.022	0.979
	Personality traits	0.195	0.028	0.283	6.887	0.000**	1.077	0.928
	Laws and regulations	−0.194	0.032	−0.287	−5.974	0.000**	1.471	0.680
	Investment education	−0.190	0.035	−0.259	−5.460	0.000**	1.436	0.696
	Typical cases	−0.173	0.034	−0.244	−5.056	0.000**	1.492	0.670
	*R² = 0.564, Adjusted R² = 0.553, F = 51.480, D-W = 1.882*							
Participation Attitude	Constant	5.063	0.411	–	12.317	0.000**	–	–
	Gender	0.046	0.115	0.019	0.398	0.691	1.043	0.959
	Age	−0.043	0.069	−0.029	−0.624	0.533	1.009	0.991
	Region	−0.050	0.114	−0.021	−0.443	0.658	1.022	0.979
	Personality traits	0.271	0.050	0.265	5.445	0.000**	1.077	0.928
	Laws and regulations	−0.176	0.057	−0.176	−3.099	0.002**	1.471	0.680
	Investment education	−0.274	0.061	−0.252	−4.487	0.000**	1.436	0.696
	Typical cases	−0.213	0.06	−0.203	−3.551	0.000**	1.492	0.670
	*R² = 0.389, Adjusted R² = 0.374, F = 25.376, D-W = 2.033*							
Subjective Norms	Constant	4.367	0.398	–	10.963	0.000**	–	–
	Gender	−0.033	0.111	−0.018	−0.300	0.765	1.043	0.959
	Age	−0.013	0.067	−0.012	−0.199	0.843	1.009	0.991
	Region	−0.011	0.110	−0.006	−0.101	0.920	1.022	0.979
	Personality traits	0.042	0.048	0.053	0.879	0.380	1.077	0.928
	Laws and regulations	−0.088	0.055	−0.113	−1.600	0.111	1.471	0.680
	Investment education	−0.021	0.059	−0.025	−0.360	0.719	1.436	0.696
	Typical cases	−0.104	0.058	−0.127	−1.789	0.075	1.492	0.670
	*R² = 0.059, Adjusted R² = 0.035, F = 2.482, D-W = 1.998*							
Perceived Behavioral Control	Constant	5.290	0.388	–	13.641	0.000**	–	–
	Gender	0.018	0.108	0.008	0.163	0.870	1.043	0.959
	Age	−0.037	0.065	−0.027	−0.576	0.565	1.009	0.991
	Region	−0.05	0.107	−0.022	−0.462	0.644	1.022	0.979
	Personality traits	0.211	0.047	0.218	4.488	0.000**	1.077	0.928
	Laws and regulations	−0.315	0.054	−0.332	−5.858	0.000**	1.471	0.680
	Investment education	−0.167	0.058	−0.162	−2.895	0.004**	1.436	0.696
	Typical cases	−0.163	0.056	−0.164	−2.880	0.004**	1.492	0.670
	*R² = 0.391, Adjusted R² = 0.376, F = 25.609, D-W = 1.901*							

Note: B = Unstandardized Regression Coefficient, S.E. = Standard Error, t-statistic = B/S.E., p = p-value, VIF = Variance Inflation Factor, R^2^ = Coefficient of Determination, F = F-statistic, D-W = Durbin-Watson Statistic.

From Table 3, the results of the multiple regression analysis reveal the complex mechanism influencing the intention to participate among victims of cryptocurrency investment fraud. In terms of model fit, the regression model for participation intention achieves an explanatory power of 55.3% (Adjusted R² = 0.553). This indicates that the model captures the major portion of the variance in the data and possesses high explanatory validity and predictive value. The regression diagnostics indicated that for all models, the Variance Inflation Factor (VIF) values were less than 5, the tolerance values were greater than 0.2, and the Durbin-Watson (D-W) values were close to 2. These results suggest that the models are free from multicollinearity issues, demonstrate good independence of residuals, and confirm that the regression results are reliable and valid.

### 4.5. The mechanism of action of factors affecting the participation intention of victims in cryptocurrency investment fraud

To further explore the mechanism of action of factors affecting the participation intention of victims in cryptocurrency. The study utilized AMOS 24.0 software to establish a structural equation model, incorporating the dependent variable, independent variables, and control variables from the multiple linear regression analysis, to conduct a structural equation analysis.

We first analyzed the fit of the structural equation model, with the fit indices presented in Appendix 7 in S1 File. The overall fit indices of the SEM indicate a good model fit, with all key fit indices meeting conventional evaluation criteria. Specifically, the χ²/df was 1.293, which is significantly below the threshold of 3, suggesting that the model’s complexity is well-controlled and it demonstrates strong structural stability. The GFI, AGFI, and RMSEA were 0.883, 0.863, and 0.032, respectively, all falling within acceptable ranges. Notably, the RMSEA of 0.032 is well below the common cutoff of 0.08, indicating smaller residuals and lower model error. Furthermore, multiple incremental fit indices—including the CFI, TLI, IFI, NFI, and RFI—all exceeded the 0.9 threshold, with the highest reaching 0.980. This demonstrates the model’s superior improvement over the baseline model, highlighting its strong explanatory power and robustness. In summary, the SEM exhibits a strong fit between the theoretical constructs and the observed data. It effectively reflects the underlying structural relationships among the variables, providing reliable statistical support for validating the research hypotheses and advancing the theoretical framework.

**Fig 3:**
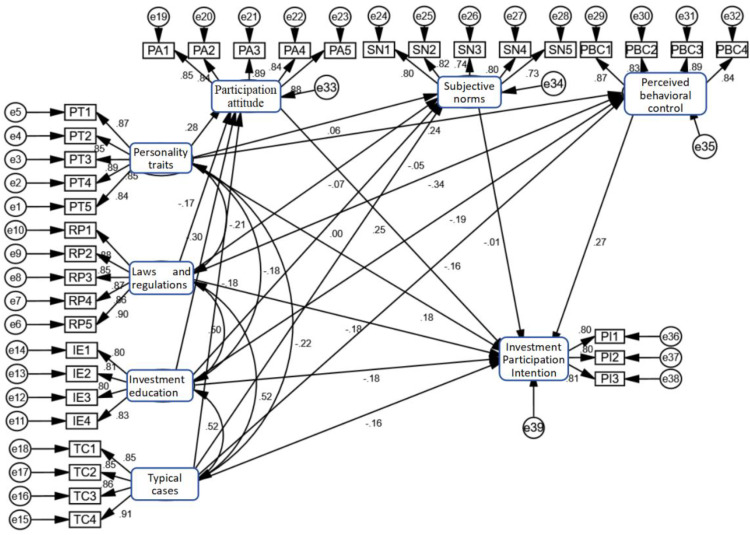
Mechanism of action of factors affecting the strength of victims’ participation intention in cryptocurrency investment fraud.

The interaction paths of various influencing factors are shown in Fig 3. As can be seen from Fig 3, the three control variables of gender, age, and region directly affect the victims’ investment participation intention. The four independent variables of laws and regulations, personality traits, investment education, and typical cases not only directly affect the victims’ investment intention but also have indirect effects through the three mediating variables of participation attitude, subjective norm, and perceived behavioral control. However, there are differences in the magnitude and significance of the impact. This is because the change in an individual’s social action intention is the result of the combined effects of various factors such as individual cognitive processes, social influences, and actual conditions, and there are multiple different paths of action.

The results of the structural equation path analysis reveal a complex network of relationships among the variables in Appendix 8 in S1 File. Personality traits exert significant positive effects on participation attitude, perceived behavioral control, and participation intention, with standardized coefficients of 0.283, 0.238, and 0.185, respectively. This indicates that individual traits play a positive role in shaping participation behavior. In contrast, regulatory policies, investment education, and typical cases have significant negative effects on these three variables, with regulatory policies showing the strongest inhibitory effect on perceived behavioral control (−0.338). Notably, the path for subjective norms did not reach statistical significance, suggesting that social pressure has limited influence in the context of this study. The positive impacts of participation attitude (0.246) and perceived behavioral control (0.266) on participation intention further confirm the applicability of the Theory of Planned Behavior. Overall, the model illustrates how individual cognition, attitudes, and external factors collectively shape the underlying mechanisms of final participation decisions, providing an empirical basis for understanding the formation of participation behavior.

To further explore the interaction paths of factors affecting the strength of victims’ investment participation intention, this paper employed a bias-corrected non-parametric percentile Bootstrap to test the mediating effects of the three mediating variables. The specific results are presented in Table 4.

**Table 4:** Standardized bootstrap mediation effect test of factors affecting the strength of victims’ participation in cryptocurrency investment fraud.

Parameter	Estimate	Lower	Upper	P
Personality traits > Investment Participation Intention direct effect	0.185	0.092	0.270	0.001
Personality traits > Investment Participation Intention total effect	0.317	0.223	0.408	0.001
Personality traits > Participation attitude > Investment Participation Intention	0.070	0.033	0.118	0.001
Personality traits > Subjective norm > Investment Participation Intention	−0.001	−0.014	0.004	0.472
Personality traits > Perceived behavioral control > Investment Participation Intention	0.063	0.028	0.115	0.001
Laws and regulations > Investment Participation Intention direct effect	−0.176	−0.290	−0.056	0.003
Laws and regulations >Investment Participation Intention total effect	−0.307	−0.427	−0.192	0.001
Laws and regulations > Participation attitude >Investment Participation Intention	−0.043	−0.097	−0.008	0.012
Laws and regulations > Subjective norm > Investment Participation Intention	0.001	−0.006	0.019	0.554
Laws and regulations > Perceived behavioral control > Investment Participation Intention	−0.090	−0.157	−0.042	0.001
Investment education > Investment Participation Intention direct effect	−0.176	−0.300	−0.049	0.008
Investment education > Investment Participation Intention total effect	−0.299	−0.425	−0.179	0.001
Investment education > Participation attitude > Investment Participation Intention	−0.073	−0.143	−0.030	0.001
Investment education > Subjective norm > Investment Participation Intention	0.000	−0.010	0.009	0.983
Investment education > Perceived behavioral control > Investment Participation Intention	−0.050	−0.111	−0.014	0.005
Typical cases > Investment Participation Intention direct effect	−0.176	−0.300	−0.049	0.008
Typical cases > Investment Participation Intention total effect	−0.299	−0.425	−0.179	0.001
Typical cases > Participation attitude > Investment Participation Intention	−0.044	−0.100	−0.010	0.011
Typical cases > Subjective norm > Investment Participation Intention	0.001	−0.006	0.018	0.607
Typical cases > Perceived behavioral control > Investment Participation Intention	−0.042	−0.104	−0.004	0.025

In Table 4, the mediation effect analysis reveals a complex mechanism through which predictors influence participation intention. Personality traits demonstrate a significant positive total effect on participation intention (0.317), exerting influence not only through a direct effect (0.185) but also through significant indirect effects mediated by participation attitude (0.070) and perceived behavioral control (0.063). This reflects the multifaceted mechanism of individual characteristics in behavioral formation. In contrast, regulatory policies (−0.307), investment education (−0.299), and typical cases (−0.299) all show strong negative total effects on participation intention. These variables directly suppress participation intention while also generating negative impacts indirectly through participation attitude and perceived behavioral control. Notably, the indirect effect of regulatory policies through perceived behavioral control (−0.090) is particularly prominent. Subjective norms did not demonstrate statistical significance in any of the mediation pathways across all variables. These conclusion are consistent with the findings presented in Appendix 8 in S1 File.

### 4.6. Standard multi-item TPB intention scale

In the standard TPB, behavioral intention is determined by three main psychological factors: behavioral attitude, subjective norm, and perceived behavioral control. The path analysis results for these three factors (Table 5) show that both attitude toward participation and perceived behavioral control have a significant positive effect on participation intention, with standardized coefficients of 0.246 and 0.266, respectively. This indicate that both play a positive role in shaping participation intention. In contrast, subjective norm do not show statistical significance, suggesting that social pressure have a limited influence in the context of this study. The results from the standard multi-item TPB intention scale are consistent with the conclusions drawn from the behavioral proxy variable.

**Table 5:** Path coefficient.

Path			Non-Standardized Path Coefficient	S.E.	C.R.	P	Standardized Path Coefficient
Participation attitude	—>	Investment Participation Intention	0.158	0.037	4.300	***	0.246
Subjective norms	—>	Investment Participation Intention	−0.012	0.042	−0.277	0.781	−0.012
Perceived behavioral control	—>	Investment Participation Intention	0.181	0.040	4.549	***	0.266

C.R. = Critical Ratio.

## 5. Conclusions

### 5.1. Results and analysis

This study investigated the factors influencing victims’ participation in cryptocurrency investment scams. It also explores the interaction mechanisms among these factors. The objective is to offer effective recommendations for crime prevention. As a novel investment method, cryptocurrency has attracted many participants. Yet, a lack of regulation has made it a fertile ground for criminal activity. Consequently, global losses from cryptocurrency fraud are rising dramatically.

Our research analyzed the causes of deception from the victim’s viewpoint. We constructed behavioral proxy variables based on relevant psychological theories. A SEM was then used for statistical analysis. This quantitative approach bypassed the psychological biases and cognitive limitations of direct measurement. It provided a more concrete and objective reflection of behavioral intent. This led to more accurate and robust estimates. It also significantly enhanced our ability to explain victims’ ultimate actions. Our findings revealed that:

First, the regression analysis yields the following results:

Personality traits have a significant positive impact on the intention to participate (β = 0.283, p < 0.001), revealing an intrinsic link between investors’ psychological characteristics and their risk behaviors. This finding reveals the significant role of personality traits in the risk decision-making process. Investors with a risk-seeking personality tend to accept higher risks in exchange for greater potential returns. Furthermore, when individuals possess traits such as confidence, curiosity, and a penchant for in-depth study, they tend to have stronger capabilities and a greater desire to realize their self-worth through investment. Consequently, their intention to participate in investing is continually driven by these individual traits and rises accordingly.

Laws and regulations exhibit a significant negative moderate effect (β = −0.287, p < 0.001), reflecting an inhibitory mechanism of the external institutional environment on individual investment decisions. A robust regulatory framework and stringent penalties can purify the investment environment, heighten investors’ awareness of criminal risks, and thereby reduce the likelihood of their involvement in fraudulent activities.

Investment education, as a key protective mechanism, demonstrates a significant negative impact on participation intention (β = −0.259, p < 0.001). Investment education enhances investors’ ability to recognize fraudulent patterns and fosters a rational investment consciousness, ultimately reducing their propensity to engage in risky investments.

The significant negative impact of typical cases on participation intention (β = −0.244, p < 0.001) reveals the preventative function of case-based warnings. By visually demonstrating the harms of fraud, these cautionary cases activate investors’ risk memory, establishing a cognitive vigilance mechanism, and reduce their propensity to engage in risky investments.

These studies are consistent with the previous researches. For example, Previous research has also indicated that the decision-making of cryptocurrency investors is driven by technical confidence (perceived behavioral control) and profit expectations (attitude) [33]. The positive effect of high-risk preference and innovation tendency on investment intention aligns with the conclusion in the financial crime field that “risk-taking personality promotes speculative behavior” [34]. The finding that typical cases suppress investment intention through risk education is consistent with the deterrent theory’s notion that “informal sanctions” (such as social warnings) play a role in crime prevention [35]. Similarly, Piquero et al. found that media reports of fraud cases can reduce the public’s willingness to participate in high-risk investments [36].

Second, the subjective norms model demonstrated low explanatory power (adjusted R^2^ = 0.035), indicating that the opinions and attitudes of others have a limited influence on investment decisions in the cryptocurrency market. This finding also, to some extent, explains the difficulties faced by financial institution staff and judicial personnel when advising victims to invest cautiously. This aligns with the conclusion that recommendations from bank managers or counter staff did not significantly influence investors’ willingness to purchase government bonds [37].

But this conclusion isn’t consistent with the traditional TPB.TPB emphasizes the key role of subjective norms (social pressure) in shaping behavioral intention, which is consistent with Ajzen’s classic conclusions [13]. However, this study found its impact to be insignificant. This may be due to the decentralized nature of cryptocurrency investment. Victims might rely more on personal judgment than on community opinions. This finding expands the applicability of the TPB. It suggests the need to incorporate a variable for “technology-mediated norms.” It also highlights the moderating role of autonomy culture. Unlike in collectivist cultures, the effect of subjective norms is weaker in individualistic environments. Therefore, cultural dimensions should be integrated into the model.

Third, the factors influencing investment intention exert dual-pathway effects: direct deterrence and indirect cognitive reshaping. From Table 4, we can see that the specific mechanisms of action of the four independent variables on the victims’ investment participation intention through mediating variables are as follows:

Personality traits have a significant positive impact on the victims’ investment participation intention through the mediating variable of investment attitude (lower limit = 0.033, upper limit = 0.118) and perceived behavioral control (lower limit = 0.028, upper limit = 0.115). Individuals who are confident and have a higher risk appetite tend to hold more positive affective and instrumental attitudes toward investment activities. Furthermore, their perceived behavioral control also increases, which can, to a certain extent, lead them to lower their guard and thereby raise their intention to invest.

Laws and regulations have a significant indirect negative effect on the victims’ investment participation intention through the mediating variables of investment attitude (lower limit = −0.097, upper limit = −0.008) and perceived behavioral control (lower limit = −0.157, upper limit = −0.042). Laws and regulations can help victims better understand the knowledge of cryptocurrency regulation and fraud crimes, clarify the composition and identification of related crimes, and thus form a higher understanding of the judgment and control required for cryptocurrency investment. This will, to some extent, reduce the victims’ enthusiasm for investment and their perceived behavioral control. The impact of Laws and regulations on Perceived Behavioral Control is particularly significant (β = −0.332, p < 0.001). This indicates that the regulatory environment establishes external constraints on risk-related decision-making by strengthening investors’ assessment of the potential consequences of their actions.

Investment education also have a significant indirect negative impact on the victims’ investment participation intention through the mediating variables of investment attitude (lower limit = −0.143, upper limit = −0.030) and perceived behavioral control (lower limit = −0.111, upper limit = −0.014). Educational investment fosters a profound comprehension of investment-related risks among individuals. They will find that new investment fraud methods are endless, and cryptocurrency investment carries greater risks. They will then realize the inadequacy of their own knowledge, abilities, and resources, and their perceived behavioral control will weaken to some extent, leading to a decline in their investment participation intention. The educational intervention exerts its greatest effect by altering participation attitude (β = −0.252, p < 0.001), which reveals the critical role of cognitive restructuring in changing risk behaviors.

Typical cases have a significant indirect negative impact on the victims’ investment participation intention through the mediating variables of investment attitude (lower limit = −0.100, upper limit = −0.010) and perceived behavioral control (lower limit = −0.104, upper limit = −0.004). Typical cases provide a real-world template, enabling victims to recognize various fraudulent schemes and investment risks. This, in turn, diminishes the psychological and physiological satisfaction that investing can bring, causes them to doubt their own knowledge and abilities, and ultimately reduces their intention to participate in such investments.

Existing literature has predominantly focused on direct path effects, whereas this study reveals the influence of indirect pathways. For example, typical cases not only provoke fear but also reconstruct behavioral logic by modifying risk perceptions. These findings provide a micro-level behavioral explanation for the “Environmental Crime Prevention” theory [38].

The overall findings support a multi-pathway influence model, demonstrating how individual cognition and external factors collectively shape participation intention through both direct and indirect pathways. This provides a theoretical foundation for constructing a multi-layered protection system. Future preventive measures should strategically strengthen regulatory frameworks, optimize investment education, and diversify warning cases. Simultaneously, intervention strategies should be designed to account for personality trait differences, ultimately establishing a comprehensive and systematic risk prevention network.

### 5.2. Theoretical implications

Grounded in the TPB, this study systematically investigates the formation mechanism of victims’ intention to participate in cryptocurrency investment fraud. Compared to existing literature, the innovation of this research lies in the construction of a multi-level, integrated analytical framework, which in turn challenges and refines the applicability of a classic theory within a specific, emerging context.

The foremost theoretical innovation is the contextual modification and boundary expansion of the TPB. Traditional TPB emphasizes the combined effect of attitude, subjective norm, and perceived behavioral control on behavioral intention, often treating the three as equally important. However, through empirical data, this study clearly demonstrates that in the unique context of cryptocurrency--characterized by its high technicality, decentralization, and anonymity-the influence of subjective norm on participation intention is significantly weakened. This finding is of great significance, as it reveals the limitations of traditional social influence theories in digitally native environments. Victims’ decision-making appears to be increasingly detached from the constraints of community pressure or the expectations of others, shifting towards a model more reliant on personal judgment, information gathering, and technical confidence. Therefore, this study does not merely apply the TPB; rather, through empirical testing, it recalibrates the weight of its core variables, clearly defining its applicability boundaries in explaining criminal behavior related to emerging technologies. This constitutes a significant contribution to the theory itself.

This study achieves a paradigmatic innovation, shifting from a “single-factor explanation” to a “systematic, integrated analysis.” Previous research has often been fragmented, either focusing on investors’ psychological traits (e.g., risk preference), concentrating on the external regulatory environment, or exploring the role of financial literacy. While valuable, these studies failed to reveal the complex interactions between various factors. In contrast, this study creatively integrates variables from four dimensions—individual psychological level (personality traits), macro-institutional level (laws and regulations), social intervention level (investment education), and informational warning level (typical cases)—into a single theoretical model. This integrated framework enables us to compare the relative influence of different types of factors within a unified logical system and to paint a panoramic picture of victims’ decision-making motivations, marking a significant leap in both methodology and cognitive depth.

This study innovates in its analytical depth, moving from the superficial correlations of “direct effects” to the internal mechanisms of “indirect effects.” We not only answer the question of “what factors influence intention” but also, through mediation analysis, systematically address “how these factors influence intention.” The research finds that the core pathway for intervention measures, such as investment education and typical cases, is not to directly suppress intention, but to indirectly achieve this by reshaping investors’“participation attitude” (e.g., reducing greed, enhancing rationality) and enhancing their “perceived behavioral control” (e.g., strengthening the ability and confidence to identify scams). This “opening of the black box” of the action mechanism makes the research conclusions more profound and persuasive, providing clear targets for subsequent precise interventions.

The systematic work of this study fills several key gaps in the current academic landscape. First, it fills the theoretical gap concerning the “psychological formation mechanism of cryptocurrency fraud victims.” Prior to this paper, research on cryptocurrency fraud was mostly limited to news reports, case analyses, or descriptions of fraudulent tactics, lacking a mature theoretical model to systematically explain why victims “willingly” participate. This study is the first to successfully apply and extend a well-established social psychological theory (TPB) to this area, providing a solid theoretical framework for this emerging social issue and advancing related research from the stage of phenomenological description to that of mechanistic inquiry. Second, it fills the research gap in “social influence theory within decentralized environments.” With the rise of Web3.0, human social interaction patterns are undergoing profound changes. To what extent do traditional social influence theories based on “communities,” “circles,” and “authority” still apply? Through empirical data, this study provides a concrete, early answer to this broad question from the domain of financial crime. It confirms that in trustless environments, the power of traditional “subjective norms” is being weakened, offering an important theoretical lead and reference for future research on human behavior in more decentralized scenarios. Third, it fills the empirical gap regarding the “effectiveness mechanisms of crime prevention interventions.” Policymakers and law enforcement agencies have been implementing various anti-fraud measures, but empirical evidence on “why they work” and “how they can work better” has been relatively scarce. Through rigorous statistical models, this study quantifies the pathways and effects of different interventions, clearly demonstrating specific transmission chains such as “regulation-risk perception,” “education-attitude reshaping,” and “cases-cognitive vigilance.” Filling this gap enables crime prevention efforts to transition from being “experience-driven” in the past to “data-driven” and “science-driven” in the future, providing an indispensable empirical cornerstone for building an efficient, precise, and low-cost crime prevention system [39].

### 5.3. Practical implications

#### 5.3.1. The survey results provide precise guidance for the selection of crime prevention strategies.

##### a. Strengthening the Promotion of Laws and regulations and Deepening Investment Education:

First, we must conduct targeted publicity on regulations and policies. According to the theory of attitude change, a person’s attitude can be influenced by changing their cognition. Publicity and education are key tools for enhancing cognition. They can therefore change people’s attitudes toward investment participation. We should diversify our communication formats. For example, we can use lively and visual methods like short videos, animations, and comics. We can also leverage channels such as live streams, social media, and online forums. It is also important to target audiences precisely. We should tailor our communication strategies for different settings. These include communities, schools, and hospitals. The strategies should be based on the specific characteristics of each audience. Furthermore, we should build multi-stakeholder partnerships. This involves collaborating with financial institutions, regulatory bodies, and law enforcement agencies. We must regularly update our content. This ensures our efforts keep pace with evolving scam tactics. This allows for continuous legal education. Through this legal publicity, we can achieve several goals. We can increase public understanding of the laws and regulations related to cryptocurrency investment. We can also improve people’s ability to identify fraudulent crimes. Ultimately, this will help curb their intention to participate in such investments.

Second, we must deepen the quality of investment education. If investment education remains superficial, it may be mistaken as a new method for wealth creation. This would ironically encourage some investors to participate blindly in cryptocurrency investments. They would do so with only a partial understanding of cryptocurrency and new financial transaction rules. Therefore, investment education must emphasize the in-depth identification of risks. We should conduct interactive activities, both online and offline. For instance, we can hold investment anti-fraud knowledge contests and simulate scam scenarios. These activities will create a more engaging and immersive experience. We should also encourage the public to share their own anti-fraud experiences and stories. This helps create a powerful and memorable cautionary impact. Furthermore, we can establish a dedicated investment advisory hotline. This would provide the public with professional consultation and assistance. We can also train a group of professional volunteers. These volunteers can go into communities to offer face-to-face advice on investment and fraud prevention. It is especially important to tailor our approach for the elderly. We must simplify the information to ensure it is easy to understand. This means using plain language and avoiding complex technical terms. We should also use visual aids like charts and pictures to help with comprehension.

##### b. Improving Personality traits and Enhancing Behavioral Control Abilities:

The refinement of personality traits and the enhancement of behavioral control skills can provide proper guidance for investment intentions. On one hand, individuals need to develop self-awareness and improve their personality traits. In our current era of artificial intelligence and a learning society, individuals should scientifically assess their own personality traits. This assessment should occur during their daily information gathering, especially when learning about cryptocurrency investments. Through theoretical study and practical training, they can improve their character qualities and avoid becoming overconfident or acting impulsively. At the same time, individuals should elevate their understanding of money, status, and success. This helps them form a sound outlook on life and values, leading to rational investment decisions.

On the other hand, individuals must continuously improve their investment skills. For those interested in cryptocurrency investment, it is not enough to simply participate in relevant educational programs. They must also enhance their financial literacy, master investment knowledge, and stay informed about investment policies. More importantly, they should gain practical experience by participating in investment activities through legitimate financial institutions. This process builds their investment competence, fosters a better attitude toward participation, and strengthens their perceived behavioral control over investing.

##### c. Enhancing the Policy Effectiveness of Typical Cases:

Psychological research shows that visual information is more memorable than auditory information, and negative information is more memorable than positive information. Therefore, methods like warning case videos can have a greater deterrent effect. These warning cases should highlight the following key points: First, explain how illegal funds from scams are laundered. This includes transfers through illegal third- and fourth-party payment platforms, transactions with online stores and shell companies, and coin-mixing transactions between cryptocurrencies. The goal is to help victims understand that once they are scammed, recovering their money is extremely difficult. Second, introduce the methods used in scams. This involves identifying keywords related to products and public opinion to stay aware of active investment gimmicks online. It also means obtaining the common scripts and phrases that scammers use during their operations. This will enable potential victims to effectively recognize new types of scams. Third, describe the scammers themselves. Advances in technology and the internet allow scammers to create fake identities, images, and locations. Victims need to understand that what they see or hear may not be real. This makes it very difficult to find the actual criminal, bring them to justice, and recover any losses after a scam occurs. By selecting typical cases that focus on the victim’s personal interests, we can create a sense of empathy and urgency. This, in turn, helps to lower their intention to invest.

#### 5.3.2. Providing a new paradigm for research on “digital crime victims”.

Cryptocurrency investment fraud is characterized by its high technology, anonymity, and cross-regional nature, which makes it difficult for traditional victim studies to explain its novel mechanisms. This paper constructs a predictive model suitable for digital crime scenarios by quantifying independent variables such as personal traits, regulatory policies, investment education, and typical cases, as well as mediating variables like investment attitudes, subjective norms, and perceived behavioral control. This provides a new path for preventing emerging crimes.

### 5.4. Limitations and future directions

While this study offers valuable insights, its limitations must be acknowledged. First, the sample suffers from selection bias. The research subjects were limited to victims who had reported the crime, thus overlooking the “silent victims”—those who did not disclose their experience due to feelings of shame. Second, the use of cross-sectional data presents causal limitations, as it cannot determine the dynamic causal relationships between variables. For instance, it is unclear whether a change in investment attitude precedes or follows the intention to participate. Third, there is a lack of cultural contextual diversity, as the data was sourced from a single country. This means the study did not compare how the model might differ under various regulatory environments, such as in China versus the United States.

Future research should address these issues by increasing the sample size and broadening its scope. Employing a variety of data collection methods and statistical techniques will help reduce the influence of variable bias and deepen the understanding of the relationships between them. Furthermore, to examine the dynamic relationships among the research variables, it is recommended to map the structural relationships between variables at different points in time. This would allow for a more detailed description of their dynamic interaction. Future studies could also deeper explore integrating technological mediation and cultural context into TPB research model.

## Supporting information

S1 File Appendix file. (DOCX)
