# Supplementary material for: Influencing factors and mechanisms of action on the participation intentions of cryptocurrency investment fraud victims—A quantitative examination from the perspective of the theory of planned behavior
Source: PLoS One. 2026 Feb 25;21(2):e0339989. doi: 10.1371/journal.pone.0339989 (PMC12935255; doi:10.1371/journal.pone.0339989)
Supplement: S1 File — (DOCX) [file pone.0339989.s001.docx]

**Appendix 1:**

**Cryptocurrency Investment Involvement Intention Survey Questionnaire**

**Dear Respondent,**

**Hi! First of all, thank you very much for taking the time to participate in this questionnaire survey. We are conducting a research on cryptocurrency investment fraud, aiming to deeply analyze the influencing factors of victims' intention to participate in investment, so that we can better prevent fraud.**

**Your opinions are very important to us and will directly help us provide targeted solutions. This questionnaire is conducted anonymously and all information will only be used for statistical analysis, ensuring that your personal information and answers are strictly confidential. Please feel free to answer according to your true thoughts and experiences.**

1. Your age is:

a. 20-40 years old b. 41-60 years old c. Over 60 years old

2. Your gender:

a. Male b. Female

3. Your place of residence is: ( ) Province

4. The proportion of cryptocurrency investment amount to personal income:(PI1)

a. 0-20% b. 20%-40% c. 40%-60% d. 60%-80% e. Over 80%

5. The proportion of cryptocurrency investment amount to personal savings:(PI2)

a. 0-20% b. 20%-40% c. 40%-60% d. 60%-80% e. Over 80%

6. The number of cryptocurrency investment projects you participate in:(PI3)

a. 1 b. 2 c. 3 d. 4 e. 5 or more

(The following questions are rated from 1 to 5: 1 means strongly disagree, 2 means disagree, 3 means uncertain, 4 means agree, 5 means strongly agree. Please mark "√" after the corresponding number.)

7. Do you think the open personality who likes to try new things has an impact on the intention to participate:(PT1)

1. 2. 3. 4. 5.

8. Do you think the sense of responsibility of working meticulously has an impact on the intention to participate:(PT2)

1. 2. 3. 4. 5.

9. Do you think the extroverted personality who likes to participate in social activities has an impact on the intention to participate:(PT3)

1. 2. 3. 4. 5.

10. Do you think the agreeable personality who easily trusts others has an impact on the intention to participate:(PT4)

1. 2. 3. 4. 5.

11. Do you think the unstable personality who is easily stressed and anxious has an impact on the intention to participate:(PT5)

1. 2. 3. 4. 5.

12. Do you think the understanding of cryptocurrency regulatory norms (such as cryptocurrency holding, trading norms, etc.) has an impact on the intention to participate:(RP1)

1. 2. 3. 4. 5.

13. Do you think the understanding of financial investment legal norms (such as financial market supervision, securities insurance futures trading regulations, contract norms, etc.) has an impact on the intention to participate:(RP2)

1. 2. 3. 4. 5.

14. Do you think the understanding of fraud crime legal norms (such as the subject of crime, the object of fraud, fraud methods, etc.) has an impact on the intention to participate:(RP3)

1. 2. 3. 4. 5.

15. Do you think the understanding of the criminal penalties for fraud crimes (such as the amount of fines, the term of imprisonment, etc.) has an impact on the intention to participate:(RP4)

1. 2. 3. 4. 5.

16. Do you think the understanding of the amount of recovered funds from fraud crimes has an impact on the intention to participate:(RP5)

1. 2. 3. 4. 5.

17. Do you think the investment education of financial institutions or financial regulatory authorities has an impact on the intention to participate:(IE1)

1. 2. 3. 4. 5.

18. Do you think the investment education of educational institutions has an impact on the intention to participate:(IE2)

1. 2. 3. 4. 5.

19. Do you think various media investment education has an impact on the intention to participate:(IE3)

1. 2. 3. 4. 5.

20. Do you think investment experience has an impact on the intention to participate:(IE4)

1. 2. 3. 4. 5.

21. Do you think that understanding cryptocurrency investment fraud cases has increased your awareness of the risks of such investments:(TC1)

1. 2. 3. 4. 5.

22. Do you think that understanding cryptocurrency investment fraud cases has increased your awareness of the methods of such investment fraud crimes:(TC2)

1. 2. 3. 4. 5.

23. Do you think that understanding cryptocurrency investment fraud cases has increased your awareness of the extent of personal losses caused by such investments:(TC3)

1. 2. 3. 4. 5.

24. Do you think that understanding cryptocurrency investment fraud cases has increased your awareness of the social impact caused by such crimes:(TC4)

1. 2. 3. 4. 5.

25. Do you think participating in cryptocurrency investment brings you physiological satisfaction:(PA1)

1. 2. 3. 4. 5.

26. Do you think participating in cryptocurrency investment brings you an increase in wealth:(PA2)

1. 2. 3. 4. 5.

27. Do you think participating in cryptocurrency investment brings you emotional satisfaction:(PA3)

1. 2. 3. 4. 5.

28. Do you think participating in cryptocurrency investment brings you satisfaction in terms of social respect:(PA4)

1. 2. 3. 4. 5.

29. Do you think participating in cryptocurrency investment brings you the realization of self-worth:(PA5)

1. 2. 3. 4. 5.

30. Do you think the opinions and attitudes of stars towards cryptocurrency investment will have an impact on you:(SN1)

1. 2. 3. 4. 5.

31. Do you think the opinions and attitudes of your relatives towards cryptocurrency investment will have an impact on you:(SN2)

1. 2. 3. 4. 5.

32. Do you think the opinions and attitudes of your friends towards cryptocurrency investment will have an impact on you:(SN3)

1. 2. 3. 4. 5.

33. Do you think the opinions and attitudes of your leaders towards cryptocurrency investment will have an impact on you:(SN4)

1. 2. 3. 4. 5.

34. Do you think the opinions and attitudes of your peers towards cryptocurrency investment will have an impact on you:(SN5)

1. 2. 3. 4. 5.

35. Do you think the degree of possession of knowledge required for cryptocurrency investment will affect the intention to participate:(PBC1)

1. 2. 3. 4. 5.

36. Do you think the ability to judge the risk of cryptocurrency investment will affect the intention to participate:(PBC2)

1. 2. 3. 4. 5.

37. Do you think the flexible network operation ability will affect the intention to participate:(PBC3)

1. 2. 3. 4. 5.

38. Do you think the ability to withstand the loss of cryptocurrency investment will affect the intention to participate:(PBC4)

1. 2. 3. 4. 5.

Thank you once again for your participation！

**Appendix 2:**

**Measurement of Constructs**

| **Constructs** | **Items** | **Factor Loadings** | AVE | CR | Cronbachα |
| --- | --- | --- | --- | --- | --- |
| Personality traits | PT1 | 0.875 | 0.744 | 0.936 | 0.935 |
|  | PT2 | 0.847 |  |  |  |
|  | PT3 | 0.894 |  |  |  |
|  | PT4 | 0.849 |  |  |  |
|  | PT5 | 0.841 |  |  |  |
| Laws and regulations | RP1 | 0.880 | 0.760 | 0.941 | 0.940 |
|  | RP2 | 0.852 |  |  |  |
|  | RP3 | 0.864 |  |  |  |
|  | RP4 | 0.856 |  |  |  |
|  | RP5 | 0.903 |  |  |  |
| Investment education | IE1 | 0.807 | 0.657 | 0.884 | 0.884 |
|  | IE2 | 0.804 |  |  |  |
|  | IE3 | 0.802 |  |  |  |
|  | IE4 | 0.828 |  |  |  |
| Typical cases | PA1 | 0.849 | 0.743 | 0.935 | 0.935 |
|  | PA2 | 0.838 |  |  |  |
|  | PA3 | 0.892 |  |  |  |
|  | PA4 | 0.841 |  |  |  |
|  | PA5 | 0.883 |  |  |  |
| Investment attitude | TC1 | 0.846 | 0.755 | 0.925 | 0.935 |
|  | TC2 | 0.849 |  |  |  |
|  | TC3 | 0.859 |  |  |  |
|  | TC4 | 0.916 |  |  |  |
| Subjective norms | SN1 | 0.799 | 0.609 | 0.885 | 0.884 |
|  | SN2 | 0.818 |  |  |  |
|  | SN3 | 0.738 |  |  |  |
|  | SN4 | 0.804 |  |  |  |
|  | SN5 | 0.730 |  |  |  |
| Perceived behavioral control | PBC1 | 0.874 | 0.738 | 0.918 | 0.917 |
|  | PBC2 | 0.832 |  |  |  |
|  | PBC3 | 0.885 |  |  |  |
|  | PBC4 | 0.840 |  |  |  |

(AVE=Average Variance Extracted, CR=Composite Reliability)

**Appendix 3:**

**Exploratory Factor Analysis**

| Name | Factor Loading Coefficients | | | | | | | Communality (Common Factor Variance) |
| --- | --- | --- | --- | --- | --- | --- | --- | --- |
|  | Factor1 | Factor2 | Factor3 | Factor4 | Factor5 | Factor6 | Factor7 |  |
| PT1 |  | 0.884 |  |  |  |  |  | 0.816 |
| PT2 |  | 0.872 |  |  |  |  |  | 0.791 |
| PT3 |  | 0.877 |  |  |  |  |  | 0.832 |
| PT4 |  | 0.852 |  |  |  |  |  | 0.779 |
| PT5 |  | 0.859 |  |  |  |  |  | 0.779 |
| RP1 | 0.835 |  |  |  |  |  |  | 0.823 |
| RP2 | 0.825 |  |  |  |  |  |  | 0.792 |
| RP3 | 0.822 |  |  |  |  |  |  | 0.802 |
| RP4 | 0.813 |  |  |  |  |  |  | 0.797 |
| RP5 | 0.860 |  |  |  |  |  |  | 0.857 |
| IE1 |  |  |  |  |  |  | 0.783 | 0.747 |
| IE2 |  |  |  |  |  |  | 0.790 | 0.738 |
| IE3 |  |  |  |  |  |  | 0.813 | 0.755 |
| IE4 |  |  |  |  |  |  | 0.791 | 0.760 |
| TC1 |  |  |  |  | 0.778 |  |  | 0.789 |
| TC2 |  |  |  |  | 0.815 |  |  | 0.799 |
| TC3 |  |  |  |  | 0.841 |  |  | 0.818 |
| TC4 |  |  |  |  | 0.866 |  |  | 0.873 |
| PA1 |  |  | 0.827 |  |  |  |  | 0.796 |
| PA2 |  |  | 0.789 |  |  |  |  | 0.773 |
| PA3 |  |  | 0.802 |  |  |  |  | 0.820 |
| PA4 |  |  | 0.809 |  |  |  |  | 0.782 |
| PA5 |  |  | 0.826 |  |  |  |  | 0.819 |
| SN1 |  |  |  | 0.838 |  |  |  | 0.715 |
| SN2 |  |  |  | 0.848 |  |  |  | 0.724 |
| SN3 |  |  |  | 0.799 |  |  |  | 0.651 |
| SN4 |  |  |  | 0.839 |  |  |  | 0.723 |
| SN5 |  |  |  | 0.797 |  |  |  | 0.653 |
| PBC1 |  |  |  |  |  | 0.807 |  | 0.824 |
| PBC2 |  |  |  |  |  | 0.805 |  | 0.787 |
| PBC3 |  |  |  |  |  | 0.800 |  | 0.825 |
| PBC4 |  |  |  |  |  | 0.780 |  | 0.778 |
| Initial Eigenvalues | 10.890 | 3.621 | 3.373 | 2.144 | 1.827 | 1.608 | 1.552 | - |
| % of Variance | 34.032% | 11.315% | 10.539% | 6.699% | 5.709% | 5.026% | 4.849% | - |
| Cumulative % | 34.032% | 45.346% | 55.886% | 62.585% | 68.294% | 73.320% | 78.169% | - |
| Rotation Sums of Squared Loadings | 4.112 | 4.096 | 3.973 | 3.471 | 3.241 | 3.094 | 3.026 | - |
| % of Variance | 12.851% | 12.801% | 12.416% | 10.847% | 10.128% | 9.670% | 9.456% | - |
| Cumulative % | 12.851% | 25.652% | 38.068% | 48.915% | 59.043% | 68.713% | 78.169% | - |

**Appendix 4:**

**KMO and Bartlett's Test**

| **Item** | | **Value** |
| --- | --- | --- |
| **KMO Measure of Sampling Adequacy** | | **0.920** |
| **Bartlett's Test of Sphericity** | **Approx. Chi-Square** | **7310.113** |
|  | **df** | **496** |
|  | **p-value** | **0.000** |

(KMO=Kaiser-Meyer-Olkin Measure of Sampling Adequacy)

**Appendix 5:**

**Discriminant Validity**

|  | Personality traits | Laws and regulations | Investment education | Typical cases | Investment attitude | Subjective norms | Perceived behavioral control |
| --- | --- | --- | --- | --- | --- | --- | --- |
| Personality traits | 0.863 |  |  |  |  |  |  |
| Laws and regulations | -0.260 | 0.872 |  |  |  |  |  |
| Investment education | -0.182 | 0.500 | 0.811 |  |  |  |  |
| Typical cases | 0.408 | -0.468 | -0.522 | 0.862 |  |  |  |
| Investment attitude | -0.222 | 0.520 | 0.520 | -0.483 | 0.869 |  |  |
| Subjective norms | 0.081 | -0.106 | -0.068 | -0.093 | 0.116 | 0.780 |  |
| Perceived behavioral control | 0.372 | -0.056 | 0.473 | -0.480 | 0.559 | 0.147 | 0.859 |

**Appendix 6:**

**Fit Indices of the Factor Models**

| **Model** | **X2** | **df** | **CFI** | **TLI** | **RMSEA** | **SRMR** |
| --- | --- | --- | --- | --- | --- | --- |
| **Seven-factor model** | 680.364 | 443 | 0.981 | 0.979 | 0.053 | 0.029 |
| **Five-factor model** | 2171.808 | 550 | 0.622 | 0.776 | 0.116 | 0.096 |
| **Four-factor model** | 2758.594 | 554 | 0.718 | 0.698 | 0.134 | 0.103 |
| **Three-factor model** | 3116.260 | 557 | 0.540 | 0.651 | 0.109 | 0.109 |
| **Two-factor model** | 3840.392 | 559 | 0.481 | 0.554 | 0.170 | 0.119 |
| **One-factor model** | 4845.161 | 560 | 0.453 | 0.419 | 0.164 | 0.141 |

(X2=Chi-Square Statistic, df=Degrees of Freedom, CFI =Comparative Fit Index, TLI =Tucker-Lewis Index, RMSEA =Root Mean Square Error of Approximation, SRMR =Standardized Root Mean Square Residual)

**Appendix 7:**

**Goodness-of-Fit Analysis of the Structural Equation Model for Factors Affecting the Strength of Victims’ Participation Intention in Cryptocurrency Investment Fraud**

| Model fitting indicators | Goodness of fit | Model fitting values |
| --- | --- | --- |
| χ^2^ | - | 691.901 |
| df | - | 535 |
| χ^2^/df | <3 | 1.293 |
| GFI | >0.8 | 0.883 |
| RMSEA | <0.10 | 0.032 |
| CFI | >0.8 | 0.980 |
| NFI | >0.8 | 0.918 |
| RFI | >0.8 | 0.908 |
| TLI | >0.8 | 0.978 |
| AGFI | >0.8 | 0.863 |
| IFI | >0.8 | 0.980 |

(GFI=Goodness-of-Fit Index, AGFI=Adjusted Goodness-of-Fit Index, NFI=Normed Fit Index, RFI=Relative Fit Index, IFI=Incremental Fit Index)

**Appendix 8:**

**Model Path Coefficients**

| Path | | | Unstandardized Coefficient | S.E. | C.R. | P | Standardized Coefficient |
| --- | --- | --- | --- | --- | --- | --- | --- |
| personality traits | —> | participation attitude | 0.282 | 0.053 | 5.340 | *** | 0.283 |
| laws and regulations | —> | participation attitude | -0.170 | 0.062 | -2.740 | 0.006 | -0.173 |
| investment education | —> | participation attitude | -0.311 | 0.070 | -4.420 | *** | -0.295 |
| typical cases | —> | participation attitude | -0.176 | 0.064 | -2.766 | 0.006 | -0.179 |
| personality traits | —> | subjective norms | 0.037 | 0.042 | 0.870 | 0.384 | 0.058 |
| laws and regulations | —> | subjective norms | -0.044 | 0.051 | -0.871 | 0.384 | -0.071 |
| investment education | —> | subjective norms | -0.002 | 0.057 | -0.035 | 0.972 | -0.003 |
| typical cases | —> | subjective norms | -0.029 | 0.052 | -0.549 | 0.583 | -0.046 |
| personality traits | —> | perceived behavioral control | 0.222 | 0.049 | 4.518 | *** | 0.238 |
| laws and regulations | —> | perceived behavioral control | -0.312 | 0.059 | -5.244 | *** | -0.338 |
| investment education | —> | perceived behavioral control | -0.186 | 0.065 | -2.842 | 0.004 | -0.188 |
| typical cases | —> | perceived behavioral control | -0.144 | 0.060 | -2.398 | 0.016 | -0.156 |
| personality traits | —> | Investment Participation Intention | 0.118 | 0.031 | 3.847 | *** | 0.185 |
| laws and regulations | —> | Investment Participation Intention | -0.111 | 0.035 | -3.129 | 0.002 | -0.176 |
| investment education | —> | Investment Participation Intention | -0.119 | 0.039 | -3.029 | 0.002 | -0.176 |
| typical cases | —> | Investment Participation Intention | -0.103 | 0.034 | -2.982 | 0.003 | -0.163 |
| participation attitude | —> | Investment Participation Intention | 0.158 | 0.037 | 4.300 | *** | 0.246 |
| subjective norms | —> | Investment Participation Intention | -0.012 | 0.042 | -0.277 | 0.781 | -0.012 |
| perceived behavioral control | —> | Investment Participation Intention | 0.181 | 0.040 | 4.549 | *** | 0.266 |
